# Process evaluation of F@ce 2.0, a team-based, person-centred intervention for rehabilitation after stroke supported by ICT

**DOI:** 10.1186/s12913-026-14628-6

**Published:** 2026-05-07

**Authors:** Kajsa Söderhielm, Jemma Hawkins, Charlotte Ytterberg, Malin Tistad, Susanne Guidetti

**Affiliations:** 1 Department of Neurobiology, Care Science & Society (NVS), Division of Occupational Therapy, Karolinska Institutet, Alfred Nobels Allé 23, Huddinge, Stockholm, 141 52 Sweden; 2 Centre for Development, Evaluation, Complexity and Implementation in Public Health Improvement (DECIPHer), Cardiff University, Cardiff, Wales, UK; 3 Department of Neurobiology, Care Science & Society (NVS), Division of Physiotherapy, Karolinska Institutet, Stockholm, Sweden; 4 Medical Unit Allied Health Professionals, Theme Women´s Health and Allied Health Professionals, Karolinska University Hospital, Stockholm, Sweden; 5 School of Health and Welfare, Dalarna University, Falun, Sweden; 6 Department of Neurobiology, Care Sciences & Society, Division of Family Medicine and Primary Care, Karolinska Institutet, Stockholm, Sweden

**Keywords:** Digital health, Mobile phone, E-health, Occupational therapy, Implementation, ADL, Activities of daily living, Goal setting

## Abstract

**Background:**

Rehabilitation after stroke is a complex process, and innovative solutions, including digital tools, have been suggested as way of meeting the challenge of limited access to services. F@ce 2.0 is a person-centred intervention for rehabilitation after stroke where goal achievement is supported by information and communication technology. Guidance for evaluation of complex interventions, such as that from the UK Medical Research Council (MRC), underlines the importance of evaluating not only the effects of an intervention but also process, such as its implementation in a clinical setting. The objective was therefore to study the implementation process of the F@ce 2.0 intervention for stroke survivors living at home as well as the interaction between the intervention and the implementation context.

**Methods:**

A process evaluation of the implementation of F@ce 2.0 was conducted, informed by the MRC guidance for process evaluations, utilising a convergent mixed methods design. Participants included stroke survivors (*n* = 39), rehabilitation team members (*n* = 53) and managers (*n* = 6). Data were collected through a web server, surveys and interviews. Descriptive statistics were used for quantitative analysis. Qualitative data were analysed using a deductive thematic approach based on the Consolidated Framework for Implementation Research.

**Results:**

Analysis revealed challenges in conducting research during the COVID-19 pandemic as well as limitations related to implementation planning in relation to the specific context. The reach of the intervention among stroke survivors was low, and fidelity issues were identified related to the teams’ lack of readiness to deliver the intervention. Analysis of teams’ perception of delivering the intervention revealed that they saw potential in using a structured instrument for person-centred goal setting and in goal reminders, but also a hesitation regarding the usability of the intervention for stroke survivors. Teams also expressed a need of further education in delivering the intervention.

**Conclusions:**

The process evaluation showed that although rehabilitation teams recognized the potential of F@ce 2.0, implementation and reach were limited, largely due to insufficient contact with the teams for both support and monitoring. Earlier involvement of the rehabilitation teams would likely have helped identify educational needs and organizational conditions necessary for successful implementation.

**Trial registration:**

ClinicalTrials.gov NCT04351178. Firs posted 2020-04-17. https://clinicaltrials.gov/study/NCT04351178.

**Supplementary Information:**

The online version contains supplementary material available at 10.1186/s12913-026-14628-6.

## Introduction

Stroke rehabilitation is a complex process involving interaction between the stroke survivor [1], the rehabilitation team, the surrounding context [2] and sometimes the stroke survivor’s close network [3, 4]. This interplay of perspectives comes to a head in the process of setting rehabilitation goals [5]. Negotiating person-centred goals has been described as challenging, and structuring the process through use of instruments such as the Canadian Occupational Performance Measure (COPM) has been suggested [1].

Another approach that is presently being explored in rehabilitation is the use of information and communication technology (ICT) to support goal achievement [6]. ICT use is one example of the rapidly developing field of digital solutions for stroke rehabilitation [7]. Developing digital health approaches has been proposed by the World Health Organization as a means for increasing equity in access to healthcare [8]. Supporting goal achievement through ICT is new to stroke rehabilitation and although intervention development is ongoing [9–13] there is a lack of well powered effect studies. Adoption of digital health solutions by the healthcare system has been slow and a need for research focusing on the cultures of change within healthcare and on the role of individuals in intervention implementation has been pointed out [14]. Voices of caution have also been raised against the risk of creating untenable work environments where healthcare professionals are not supported in adapting to new technologies [15]. In recent years, concerns have also been raised about the risk of digital exclusion among disadvantaged groups in society [16]. Within rehabilitation research, particular attention has been given to stroke survivors living with aphasia and/or more severe cognitive impairment [17]. Recommendations have therefore been made to study not only the outcomes of digital rehabilitation but to analyse implementation processes to be able to identify crucial factors for success [18]. This includes consideration of the possible mechanisms of change of rehabilitation interventions, i.e. causal links between intervention components and outcomes, as well as the proposed implementation context. Moreover, the multifaceted nature of rehabilitation calls for consideration of all stakeholder perspectives when developing and implementing new interventions. To support development of interventions that are feasible in real life settings, it is crucial to investigate how an intervention is experienced by different stakeholders [19]. The UK Medical Research Council (MRC) and the National Institute for Health Research (NIHR) guidance for developing and evaluating complex interventions details an iterative process for exploring these factors and clarifying the theorised link between intervention activities and effects, including the use of visualisations [19]. The MRC/NHIR guidance advocates a broadening of the concept of evaluation, moving beyond a focus on outcomes for primary recipients by conducting a process evaluation describing interaction between the context, the implementation process and the proposed mechanisms of change [19]. Process evaluation allows researchers to seek an understanding of why an intervention has worked or failed, i.e. whether effects can plausibly be attributed to the proposed mechanisms of change and what contextual factors were pivotal for implementation [20]. The MRC guidance for process evaluation [20] is an overarching framework and can be used in combination with other frameworks to support operationalisation of key concepts, such as the Consolidated Framework for Implementation Research (CFIR) [21].

The present study is a process evaluation of F@ce 2.0, an intervention where person- centred rehabilitation after stroke in the home setting is supported by ICT [22]. The intervention has been developed via an iterative process including both healthcare professionals, stroke survivors and significant others in Sweden and Uganda [6, 23, 24]. A recent evaluation investigating effects of F@ce 2.0 on activity performance and satisfaction with activity among stroke survivors did not find any differences between the intervention group and a control group receiving rehabilitation as usual *(Söderhielm et al.*,* submitted).* To explore the implementation of F@ce 2.0 as well as the interaction between the intervention and the context of delivery, a process evaluation was conducted alongside the effectiveness evaluation. This study reports the findings of that process evaluation.

### Objective

The aim was to study the implementation process of the F@ce 2.0 intervention for stroke survivors living at home as well as the interaction between the intervention and the implementation context.

The specific research questions were:

1. What contextual factors influenced the implementation of the intervention and the extent to which its intended mechanisms of change were realised?
2. What were the characteristics of the implementation process and outcomes?
3. What were rehabilitation teams’ experiences of delivering the intervention components?

## Methods

### Research design

**Fig. 1:**
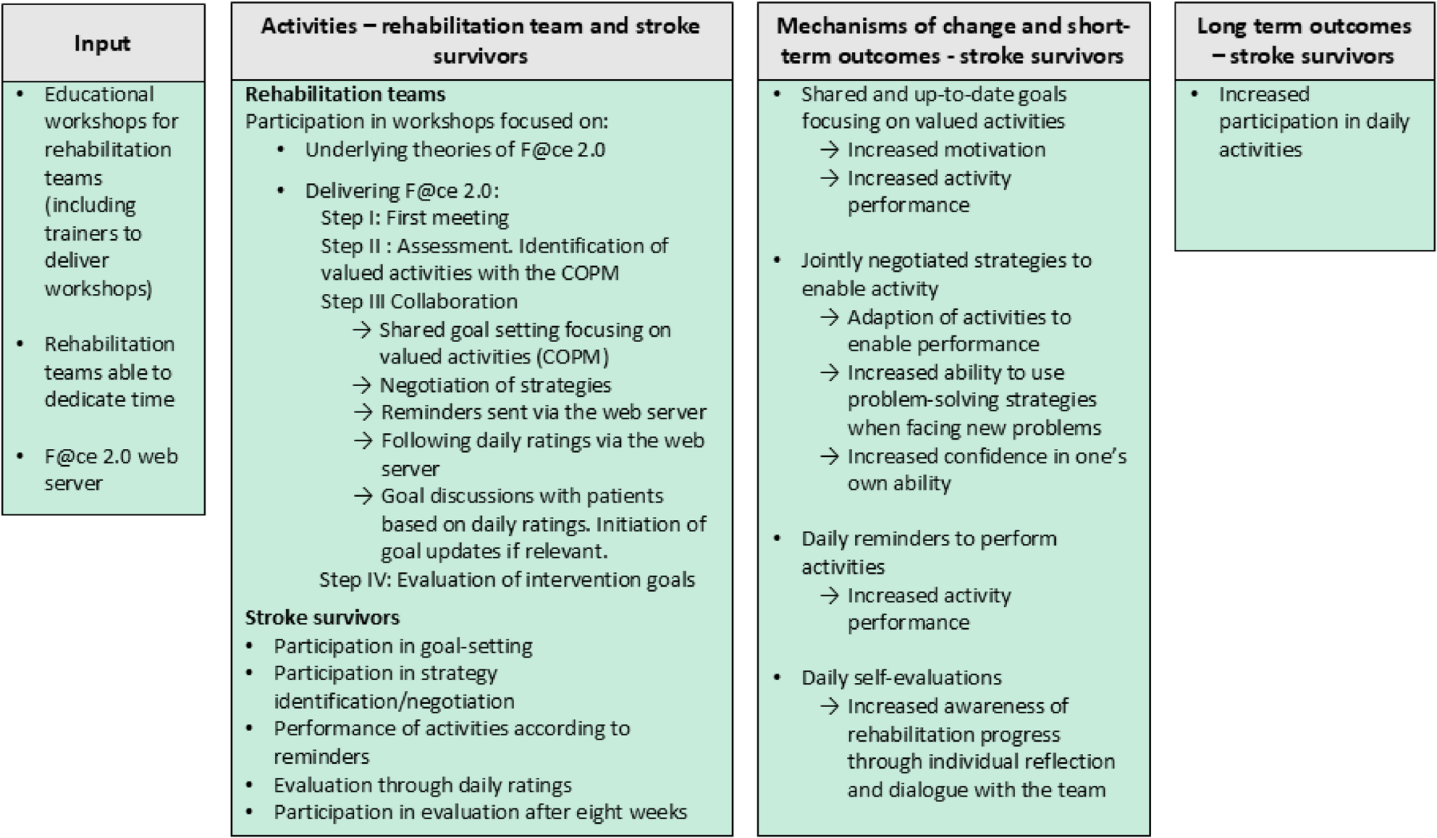
Logic model of the F@ce 2.0 intervention

The study design, data collection and initial analysis were informed by the MRC guidance for process evaluation [20] using a convergent mixed methods design [25]. To further structure the results in answer to research questions 1–2, the Consolidated Framework for Implementation Research (CFIR) [21] was applied. According to CFIR “Context, broadly defined as everything outside the EBI [Evidence Based Intervention, *authors comment*], includes the dynamic and diverse array of forces working for or against implementation efforts” [21]. To study experiences of delivering the intervention components (research question 3), an experiential analysis of interviews with rehabilitation teams was conducted based on the intervention components and the proposed mechanisms of change outlined in the logic model of the intervention (Fig. 1).

### Study setting

The study was nested within a larger project investigating the F@ce 2.0 intervention including an effectiveness study (*Söderhielm et al.*,* submitted*) as well as studies exploring experiences of the intervention from the perspective of stroke survivors [26] and family members [27]. During the project period (January 2021 to April 2023), the F@ce 2.0 intervention was implemented by rehabilitation teams spread out geographically, representing both urban and rural areas in Sweden. Recruitment of stroke survivors was conducted between February 2021 and October 2022.

### Participants

Participants included rehabilitation teams delivering the F@ce 2.0 intervention during the project period, as well as team managers and stroke survivors participating in the intervention.

#### Rehabilitation teams and managers

Seven rehabilitation teams were recruited through convenience sampling in the three Swedish healthcare regions where researchers were operating. Inclusion criteria for teams were that they offered home-based rehabilitation, that stroke survivors was one of their major patient groups, that the team included at least one physiotherapist and one occupational therapist, and that they were willing to implement and deliver F@ce 2.0. Inclusion criteria for health professionals were that they were members of the teams included in the study, whereas managers held the operational responsibility for those teams. In total, 53 healthcare professionals participated in the study, 46 women (mean age 46) and seven men (mean age 33). Experiences of working with stroke rehabilitation varied between 0.5 and 35 years, with a mean experience of 10 years, and 72% of rehabilitation team members stating that they had participated in some kind of specialist stroke training (see Table 1). Teams varied in size, ranging from four to 10 healthcare professionals. All teams included an occupational therapist and a physiotherapist, while other professions represented were speech-language therapists (five teams), social workers (five teams), physicians (two teams), nurses (one team), and dietitians (one team). The mission of teams varied, with two teams servicing stroke survivors only, while the others saw both stroke survivors and people with other neurological diagnoses. Services consisted of a mix of home-based and outpatient services with the initial contact typically held in the home of the stroke survivor. All teams stated that their usual rehabilitation process followed the structure of setting goals and coming up with a rehabilitation plan and that the number of home visits varied according to the need of the patient. For five of the teams (B and D-G), the length of rehabilitation was flexible, while the other two (A and C) had a time limited intervention period of about one month. Team A did, however, state having some degree of flexibility.

Of the six managers participating in the study (four women and two men), three had held their positions for fewer than two years. All managers were responsible for employees other than the team participating in F@ce 2.0, with a range of 17–50 employees per manager.

**Table 1:** Description of rehabilitation teams

Team	MISSION AND context	Professions in the team	Home visits/day per healthcare professional on average
A	Mixed diagnoses Early Supported home rehabilitation. Urban (small town, ca. 13 000 inhabitants) and rural setting	2 Occupational therapists 2 Physiotherapists 1 Speech language therapist (consultant)	1–4 visits (Length depends on patients’ needs. First visit can take up to 120 min.)
B	Stroke diagnosis. Early Supported home rehabilitation. Urban (small town, ca. 40 000 inhabitants) and rural setting	1 Medical social worker 1 Nurse 2 Occupational therapists 1 Physician 2 Physiotherapists 1 Speech language therapist (consultant)	2–3 visits (approx. 60–120 min / visit)
C	Stroke diagnosis Early Supported home rehabilitation Urban (medium town, ca. 90 000 inhabitants) and rural setting	1 Dietician 1 Medical social worker 1 Nurse 2 Occupational Therapists 1 Physician 2 Physiotherapists 1 Rehab Assistant 1 Speech language therapist	2–3 visits (approx. 60–90 min / visit)
D	Mixed neurological diagnoses Home rehabilitation Urban (ca. 1 million inhabitants) and suburban setting	2 Medical social workers 3 Occupational therapists 3 Physiotherapist 3 Speech therapists	4 visits (approx. 45–60 min / visit)
E	Mixed neurological diagnoses Home rehabilitation Urban setting (ca. 1 million inhabitants)	1 Medical social worker 2 Occupational therapists 3 Physiotherapists 1 Speech therapist	4 visits (approx. 45–60 min / visit)
F	Mixed neurological diagnoses Home rehabilitation Urban setting (ca. 1 million inhabitants)	1 Medical social worker 2 Occupational therapists 2 Physiotherapists 1 Speech therapist	3–5 visits (approx. 45–60 min / visit)
G	Mixed neurological diagnoses. Home rehabilitation Urban (ca. 1 million inhabitants) and suburban setting	1 Medical social worker 2 Occupational therapists 3 Physiotherapists 2 Speech therapists	4–5 visits (approx. 45–60 min / visit)

#### Stroke survivors

Stroke survivors participating in the F@ce 2.0 project were living at home and received services from one of the participating teams. Teams were instructed to inform all new patients who fulfilled the inclusion criteria about the study. If a patient was interested, personal data was communicated to the research team who handled recruitment. Inclusion criteria were (A) diagnosed with stroke, (B) receiving rehabilitation from one of the participating rehabilitation teams, (C) able to participate in an 8-week intervention, (D) able to participate in formulating activity goals, (E) self-reported ability to use a mobile phone. Of the 100 stroke survivors included in the study, 45 were recruited to the intervention group. In the intervention group, four opted to leave the study, and two were deceased before starting the intervention resulting in a total of 39 participants (3–8 per intervention team). Intervention participants (23 women, 16 men) had a mean age of 72 years (range 34–93) with a majority (37 participants) having a mild or very mild stroke according to the Barthel Index [28]. For further participant data, see Table 2.

**Table 2:** Demographic data, stroke survivors

Demographic data, stroke survivors (*n* = 39)	
Age in years, mean (SD), range	72 (12), 34–93
Men/Women, n (%)	23/16 (59/41)
Mobile phone use	
Limited (calls only or reads but doesn’t send SMSs) (%)	14 (36)
Reads and sends SMS (%)	23 (59)
Non-user (%)	2 (5)
Cohabiting, n (%)	30 (77)
Receives assistance from home services, n (%)	19 (49)
Days since stroke at inclusion	
Mean (SD)	114 (92)
Median (IQR)	79 (110)
Stroke severity Barthel index (0-100), n (%)	
Very mild (95–100) (%)	18 (46)
Mild (50–94) (%)	19 (49)
Moderate or severe (< 50) (%)	2 (5)

### The F@ce 2.0 program

The F@ce 2.0 program entailed the F@ce 2.0 intervention, workshops for rehabilitation teams, and design of the F@ce 2.0 web server.

#### The F@ce 2.0 intervention

F@ce 2.0 is an intervention for rehabilitation after stroke, aiming to increase performance in daily activities and participation in everyday life for stroke survivors [22]. The F@ce 2.0 intervention represents an extension of a Client Centred ADL-intervention (CADL) which was developed based on theories on the process of change after stroke and refined through collaborative workshops with occupational therapists [29]. Evaluation of CADL provided rational for a redesign into a team-based intervention [30]. Furthermore, an ICT support component was added as a tool for increasing sharing and transparency during the rehabilitation process [23]. The development of F@ce 2.0 was conducted both in Sweden and Uganda and testing in both settings revealed that stroke survivors found the ICT component feasible and helpful [6, 23]. Two general strategies are applied in the intervention: (1) using the person’s lived experience as a point of departure through setting person-centred goals and (2) enabling significant experiences to be gained from performing valued daily activities. The following paragraph describes the details of the F@ce 2.0 intervention along with the proposed mechanisms of change which can also be found in the logic model (see Fig. 1). The logic model was developed by the research team and informed by previous studies regarding F@ce as well as CADL, including data on experiences of the interventions [6, 23, 24, 29, 31].

F@ce 2.0 follows four steps: Face-to-face meeting, Assessment, Collaboration and Evaluation [23]. During step I, *Face-to-face meeting*, the rehabilitation team starts building a therapeutic relationship with the person, getting to know abilities, roles and habits. Involvement by significant others is encouraged. During step II, *Assessment*, the rehabilitation team engages in dialogue to reach a shared understanding of the impacts of stroke specific for that person. As part of this, an interview based on the COPM [32] is carried out to identify daily activities that the person wants and needs to perform during rehabilitation. The focus of rehabilitation will thus be on activities that bring meaning and purpose to their everyday life [33]. Performance of each activity and satisfaction with that performance is scored using the 1–10 scale of the COPM. The proposed mechanism of change for step 1 and 2 is that person-centred goal setting focused on daily activities will lead to increased motivation and performance of rehabilitation activities due to personal relevance. To enable person-centred goal setting, the importance of listening and coming to a shared understanding of the consequences of stroke is underlined during training workshops. In step III, C*ollaboration*, three person-centred goals are negotiated between the team and the stroke survivor based on the identified daily activities. The team and the stroke survivor then engage in a discussion to find strategies for reaching the goals. Here, the global cognitive strategy of Goal-Plan-Do-Check [34] is utilised. Collaboration further entails ICT support. Goals and strategies are registered on the F@ce 2.0 web server that sends out daily SMSs at two time points, one in the morning as a reminder of goals and one in the afternoon or evening requesting the person with stroke to rate their perceived level of success in the activity (goal) during the day on a scale from 1 (not so good) to 5 (worked very well). Ratings are accessible to the rehabilitation team and stroke survivors through a web server. The team also receives an SMS with a red flag when ratings are low (1–2) or when no rating is performed, indicating a need to update goals or strategies. The proposed mechanisms of change for step III of the intervention are that identification and formulation of strategies for the chosen activities will enable activity performance, but also strengthen problem solving and self-efficacy, creating a readiness for facing future challenges related to daily activities. Daily reminders to perform the chosen activities is hypothesised to increase activity performance through motivation. Daily self-evaluations are theorised to increase awareness through own reflections and through discussions with the rehabilitation team. The ratings further provide a way for the team to monitor stroke survivors’ daily progress (by accessing the F@ce 2.0 web server) and to adjust goals and strategies when low rating indicate that this is needed. Step IV, *Evaluation* of the goals is carried out at the end of the eight-week intervention using the COPM, and a plan is made for continued rehabilitation. This follow-up meeting is either held in the patient’s home or at the outpatient clinic.

#### F@ce 2.0 web server

The F@ce 2.0 web server had separate sections for stroke survivors and teams, thus functioning both as a system for enabling the ICT component and as an implementation strategy by providing access to educational materials. Rehabilitation teams registered goals and strategies on the web server and could log in to follow stroke survivors’ daily ratings. Although the main ICT-tool for stroke survivors was the daily SMSs, they could also log in to the web server to access their goals, strategies and daily ratings.

#### Implementation strategies

Implementation strategies are presented in a chronological order according to the Expert Recommendations for Implementing Change (ERIC) [35] and with the addition of the code *communication* suggested by Boyd and Powell [36]. Codes are presented in italics.

During the process of recruiting teams, *local consensus discussions* were held with managers to ensure that the intervention was suitable for clinical development in their context. *Educational materials* were *developed* at the start of the project and consisted of four pre-intervention workshops. The workshop format had been tested in a feasibility study of F@ce [23], but the COVID-19 outbreak necessitated an adaptation to an online format. The four workshops (eight hours in total, spread out over four weeks) focused on the theoretical background of the components of F@ce 2.0 (workshop 1), using the COPM in goal setting (workshop 2), delivering F@ce 2.0 and using the web server (workshop 3), and integration of F@ce 2.0 with rehabilitation as usual (workshop 4). To *make training dynamic*, workshops were designed with a mix of lectures and discussions with practise tasks between the sessions. Workshops were planned so that teams from different geographical locations would meet, aiming to *create a learning collaborative*. All *educational materials* from the workshops were also *distributed* to the teams via the F@ce 2.0 web server to allow for later access. Teams were offered a refresher workshop if they felt that this was needed. In addition, a reminder card with the intervention components was sent to all teams via post.

To *provide clinical supervision* and *build a coalition* with the teams, researchers ensured regular contact with the designated contact person in each rehabilitation team. These contacts also allowed researchers to discuss recruitment of potential participants. Due to the COVID-19 pandemic, contacts were restricted to telephone or e-mail. To strengthen the relationship, in-person visits to each team were conducted as soon as circumstances allowed (in December 2021). *Communication* with the teams was also upheld through a regular newsletter distributed via e-mail.

### Data collection

**Fig. 2:**
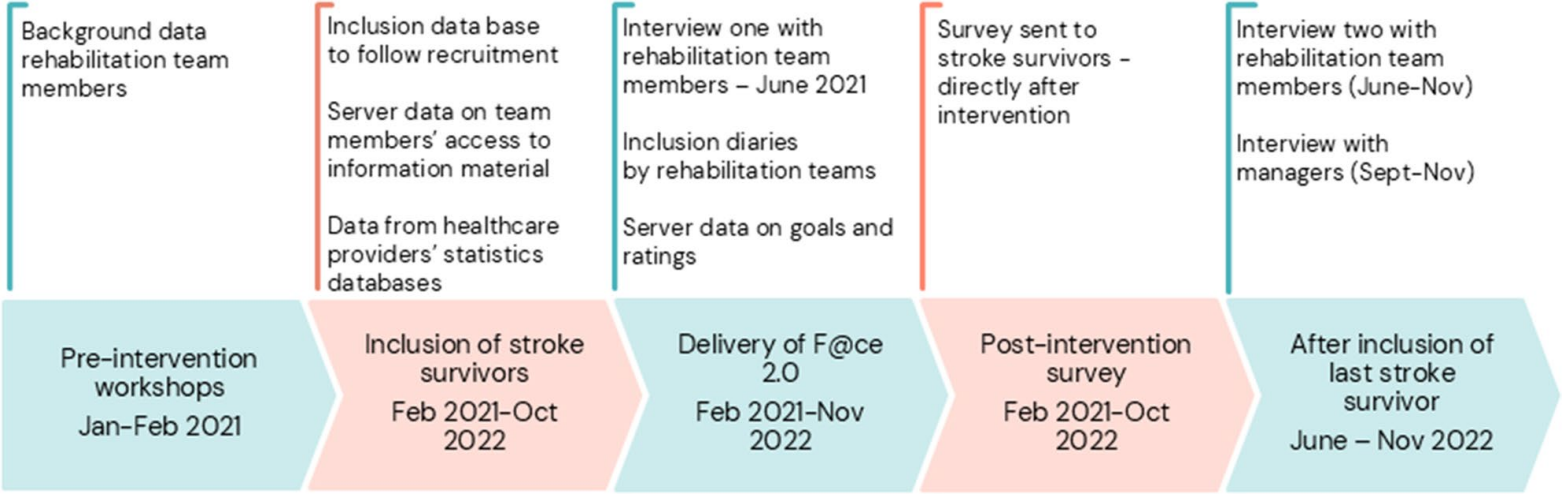
Timeline of data collection

Data were collected from participants with stroke, rehabilitation teams, team managers and from databases based on the MRC guidance for process evaluation [20] (see Table 3). The timeline of data collection is presented in Fig. 2.

**Table 3:** Data overview

Domain	Assessment	Data collection method
Context (Research question 1)		
	Experience of contextual factors affecting the implementation of F@ce 2.0	Team focus group interviews Manager interviews (individual)
Implementation (Research question 2)		
Implementation strategies	How the implementation strategies were received	Team focus group interviews Manager interviews F@ce 2.0 web server data
Fidelity	Intervention delivery as intended	Team focus group interviews Manager interviews Survey to stroke survivors F@ce 2.0 web server data (frequency of access and goal updates)
Adaptation	Description of intervention adaptations	Team focus group interviews
Dose	Stroke survivors’ engagement with daily ratings	F@ce 2.0 web server data
Reach	To which extent the intervention reached the designated participants	Inclusion database Team focus group interviews Inclusion diary (teams) Healthcare providers’ statistics databases
Mechanisms of change (Research question 3)		
	Teams’ experience of delivering the intervention components.	Team focus group interviews Manager interviews

The structure of the table follows the MRC guidance for process evaluation [20]

#### Interviews

All interview guides were developed originally for this study and were based on the MRC guidance for process evaluations of complex interventions [20] (see supplement 1). Focus group interviews with rehabilitation teams were conducted at two time points, aiming to capture experiences from the workshops and of the early phase of implementing F@ce 2.0 as well as reflections after having more extensive experience of the intervention. Interviews with teams were conducted without the presence of managers to allow them to speak freely. Interviews were held four months post start of inclusion of stroke survivors (4/7 teams) and again 16–21 months post implementation start when inclusion of stroke survivors had been concluded (7/7 teams). Interviews lasted between 40 min and one hour and were conducted by two experienced researchers, of whom one was responsible for all interviews at the first time point, while the other conducted all but one interview at the second time point. Managers were interviewed individually to increase the understanding of their role as part of the context for implementation. Individual interviews with managers (6/7) were conducted in parallel to the second rounds of team interviews by the same researcher. Interviews followed semi-structured interview guides (separate for teams and managers and for focus group one and two). Interview guides were developed by the research team and were based on the steps of the intervention as well as the teams’ experiences of participating in the workshops and of delivering the intervention.

#### Rehabilitation survey

To allow for evaluating fidelity to the intervention components from the perspective of stroke survivors, a study specific survey was developed by the research team (*supplement 2*). Questions were aimed at capturing elements of implementation fidelity via stroke survivors’ perception of goal setting being activity-based and set in collaboration with the team. The survey consisted of six multiple choice items rated on a Likert scale from one to five and one free text item asking about their specific goals. The survey was designed bearing the target group of stroke survivors in mind, keeping questions short and retaining the same grading scale for all questions. The survey was sent to all participants via mail after eight weeks of whom 30/39 replied.

#### Other process data

To allow for a detailed description of participants, background data on rehabilitation team members were collected through a survey that was sent out in connection to the workshops. To enable analysis of the implementation process, specifically fidelity, implementation strategies and dose, quantitative data were collected in multiple ways. Data on stroke survivors’ and healthcare professionals’ engagement with the ICT component were extracted from the F@ce 2.0 web server. Data from the web server were available for 35 stroke survivors and for all rehabilitation teams. Data regarding the inclusion process and eligible participants were compiled from a survey specific inclusion database, from logbooks kept by the rehabilitation teams during the inclusion period and when possible, from healthcare providers’ statistics databases detailing inflow of patients.

### Data analysis

#### Qualitative data

Analysis of interview material was deductive following a thematic approach [37]. All interviews were transcribed verbatim by KS. In the first step of analysis, data were coded deductively using an analysis frame focusing on the context, implementation and mechanisms of change. The analysis frame *(supplement3*) was developed by KS, SG and JH and was based on the MRC guidance for process evaluation [20]. Further thematic analysis of codes was conducted within each of the three domains to identify patterns related to the study questions. KS performed initial coding which was then discussed and further refined in meetings with JH and SG.

To further refine analysis and allow for a more structured description of results for research questions 1 and 2, the CFIR [38] was applied along with the outcomes addendum [21]. To operationalise the construct of Implementation outcomes to address research question 2, the original concepts from the MRC guidance for process evaluation [20] were kept as data collection has been planned following this structure. The concepts applied under Implementation outcomes were thus fidelity (whether the intervention was delivered as intended), adaptations (changes made to the intervention to better suit the context), dose received (to what degree ratings were performed by stroke survivors) and reach [20].

To address research question 3, data regarding healthcare professionals’ experience of implementing the intervention, including their perceptions of the intervention components, were analysed deductively according to their presentation in the logic model (Fig. 1). All qualitative analysis was conducted using Microsoft Excel.

#### Quantitative data

Descriptive analyses of data from the survey, the F@ce 2.0 web server, the inclusion data base and healthcare providers’ statistics databases were conducted. These data were connected to the implementation process (research question 2) and assessed fidelity (frequency of rehabilitation team access to the web server and mean scores on survey data regarding rehabilitation goals and involvement in goal setting), dose received (frequency of stroke survivor responses) and reach (time flow analysis of the inclusion process). The analyses were performed using Microsoft Excel.

## Results

### Context

Analysis of context was guided by research question 1, i.e. “What contextual factors influenced the implementation of the intervention and the extent to which its intended mechanisms of change were realised?”

This section presents findings related to context and implementation processes using the domains of the Consolidated Framework for Implementation Research (CFIR) [38] as headings, subdomains are marked in the text using *italics*. For an overview of domains and subdomains used, please see supplement 4.

### Innovation

The innovation domain of CFIR describes the characteristics of the innovation with a focus on factors potentially affecting implementation.

Although the rehabilitation teams participating in the study felt that the *design* of F@ce 2.0 was promising, concerns were raised regarding its suitability for their context. On example of this was that rating (COPM + daily goal rating) was seen as potentially too cognitively challenging for some stroke survivors. Two teams further expressed a concern that even if strategy use was enhanced by F@ce 2.0, stroke survivors would still need guidance from the team to come up with adequate strategies. Considering this, the idea of F@ce 2.0 possibly being better suited for other diagnoses was put forward during some of the interviews. A particular concern regarding its *complexity* was the use of the COPM which was seen as challenging and time consuming. Teams did, however, see a *relative advantage* of using the COPM in relation to identify personally relevant goals. A further advantage discussed by the rehabilitation teams was the potential of reminders as motivators to get activities and training done as well as a possible support for family members who often take responsibility for training in the home environment. Reflections from the teams thus indicate that realisation of the mechanisms of self-reflection and self-led problem solving may be affected by cognitive impairment after stroke.

### Outer setting

The domain of outer setting in CFIR pertains to the larger system within which the intervention was implemented. In the analysis, the subdomains of critical incidents and financing were identified as crucial. Regarding *critical incidents*, the COVID-19 pandemic was highlighted both by managers and teams. Undertaking the implementation of F@ce 2.0 while handling reorganisation and adaptions related to the pandemic was described as highly demanding.

*It’s everything from the pandemic and like restrictions and other things to adapt to. We have had a lot*,* a lot has been going on for us* Manager, team D.

*Financing* differed between teams since the implementation of F@ce 2.0 was carried out within regular operations with no extra resources, and thus reliant on the financial systems in the respective regions. This meant that the amount of time spent during home visits differed between teams, potentially affecting the proposed mechanism of increasing motivation through person-centred goal setting.

### Inner setting

The domain of inner setting in CFIR describes the relation between the innovation and the setting in which it is implemented.

The F@ce 2.0 intervention follows a generic structure for rehabilitation and regarding *compatibility*, teams reported that the intervention process was similar to their usual practice of setting goals and planning rehabilitation in collaboration with stroke survivors. The degree of *compatibility* between F@ce 2.0 and usual practice differed for the respective intervention components. Goal setting focused on daily activities was already applied by the teams, but they had not previously used the COPM. The notion of it being an occupational therapy instrument and the extra burden this meant for occupational therapists was also highlighted. The ICT-component was new, both in terms of the form (using the web server to register and communicate about goals) and in terms of function (daily team-patient contact) and was described as an additional task requiring attention and time. “*Well*,* that’s what has been a bit of extra work. This thing of registering* [*the goals*]. *If we had gone in more often*,* I guess we would have*,* maybe we would have gotten a bit more of a routine*”. Team B.

The aspect of *work infrastructure* was discussed in various ways as barriers to implementation of F@ce 2.0. Firstly, organisational demands on managers affected their involvement. Managers were typically responsible for more than one team and stated having limited time for hands-on leadership. All teams but one described this lack of resources in terms of support from managers and depicted a process where they were left alone with prioritising and making all decisions related to the project.

*We have been managing things on our own I would say. I would say that we have received neither support nor involvement [from the manager] and maybe we didn’t expect that either.* Team F

*Work infrastructure* was also discussed in terms of staffing. Managers talked about the problem of finding and keeping competent staff while team members described that existing resources did not allow for extra assignments, such as introducing new colleagues to the F@ce 2.0 intervention. Resources for rehabilitation development were also discussed against the backdrop of *relative priority.* Most teams portrayed the complexity of continuously striving to improve rehabilitation practices while at the same time experiencing time constraints, an aspect that was particularly prominent during COVID-19. Mangers’ perception of teams’ *learning-centredness* differed and was discussed against the backdrop of the busy everyday practice.

### Individuals

The individual’s domain of CFIR focuses on the roles and characteristics of people involved in the implementation process.

Analysis of the interview data displayed a lack of clear *roles* in the implementation process. Although each team had a designated contact person, the responsibility associated with this role was perceived as vague. In general, teams were unclear about who was supposed to lead the implementation of F@ce 2.0 and some teams conveyed a feeling of having been left alone with the process.

Lack of *capability* was discussed by the team in relation to using the web server where some team felt that they would have gained from a more thorough introduction. Furthermore, the teams conveyed that they did not feel prepared to use the COPM. *I think it’s like a completely new way. We haven’t used the COPM before so It’s also about learning something new and that the team has to learn this and we have had very few so it’s really hard to evaluate with so few patients. I mean it’s about us learning to handle this [COPM]*. *Team C*. Reasoning around the COPM also pertained to the concept of *opportunity*, related to the added time needed for conducting the COPM-interview. The aspect of *opportunity* is also applicable regarding the roles of leaders in clinical development, although this issue was lifted in a few interviews only. Managers were expected to lead the team according to their usual routine and no specific adjustments in relation to F@ce 2.0 were required. In hindsight, a few of them felt that the level of engagement from their side in facilitating implementation of F@ce 2.0 may have been unsatisfactory in relation to the teams’ implicit expectation of being supported. Teams’ *motivation* to participate in the implementation varied, with a positive factor lifted being the change in quality of rehabilitation goals related to use of the COPM. Some teams did, however, report not having been involved in the decision to participate in F@ce 2.0 and felt that the intervention was not suited to their context.

### Implementation process and outcomes

Analysis of the implementation process was guided by research question 2, i.e. What were the characteristics of the implementation process and outcomes?

#### Implementation process

Analysis of team interviews revealed challenges related to the tailoring of the workshops for rehabilitation teams *(tailoring strategies).* The workshops were planned to be interactive and allowed for engagement by all team member but access to computers was limited and team members sometimes had to sit together in one room. This influenced discussions negatively, rendering it difficult for researchers to facilitate active participation. It also made hands-on-demonstrations more complex. In the interviews, teams conveyed a feeling of workshops being too focused on theories, leaving limited space for the practicalities of intervention delivery. Some team members reported being inadequately prepared for delivering F@ce 2.0. An example of this was uncertainty of what stroke survivors were supposed to rate in their daily SMS reply. “*This question of is it a goal or is it a strategy… And what should be put in the SMS. Is it the strategy or the goal*” *Team A*. The interviews further indicated that the problem-solving component of F@ce 2.0 would have needed to be more specifically addressed during the workshops since teams did not feel prepared to use it with stroke survivors.

**Fig. 3:**
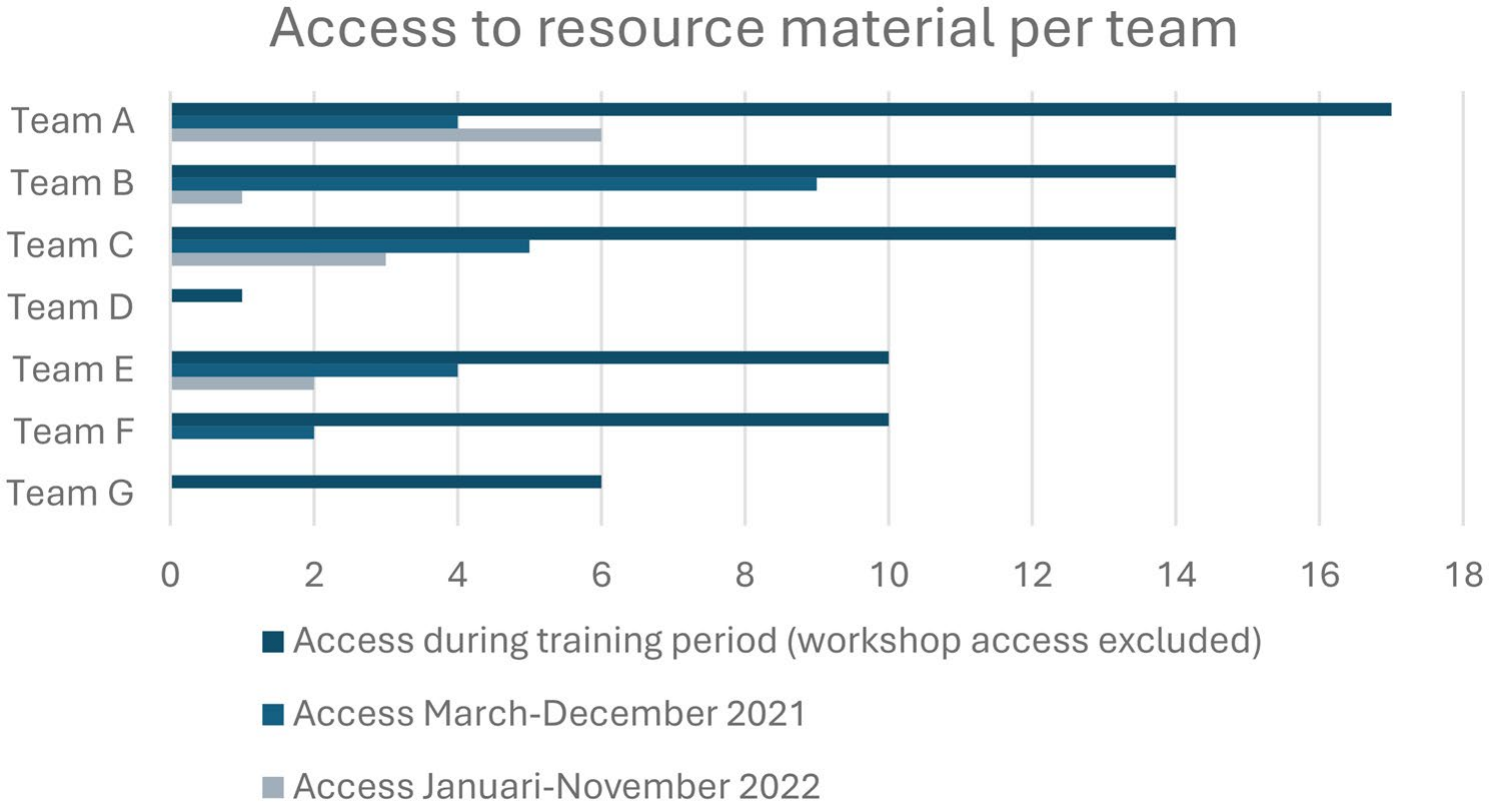
Access to resource material per team (number of times material was accessed)

Despite having questions about the intervention, teams’ web server access to rehearse or clarify information was limited *(reflecting and evaluating)*. In total, 31 (58%) of the team members accessed information material on the web server at some point during the study. Access varied between teams from once to 28 times and mainly occurred during and just after the workshop period, with only occasional visits during the second year of data collection *(see* Fig. 3*).*

In terms of *planning*, a weakness was that the role of managers in driving the implementation process was not made clear. A further limitation was not anticipating and planning for staff turnover as interviews revealed that teams lacked routines for introducing the intervention to new staff.

In terms of *engaging* the teams in the implementation process a theorised strategy to foster engagement was the regular telephone contact between each rehabilitation team and the designated contact person. From the interviews, these phone calls seem to have mainly focused on recruitment and teams conveyed a feeling of obligation to include participants. This led to a sense of stress which was communicated in all interviews, even by teams who stated that the overall contact with the researcher was characterised by a positive and helpful atmosphere. “*Sometimes I’ve had a bit of a guilty conscience. No we don’t have anyone [to include] this week either*” Team B.

#### Implementation outcomes

Implementation outcomes were assessed using both qualitative and quantitative data. As data collection regarding implementation outcomes was guided by the concepts of the MRC guidance for process evaluation, these headings are used rather than the subdomains of the CFIR Outcomes Addendum. An overview of implementation outcomes can be found in supplement 5.

#### Fidelity

Focusing on daily activities and involving the stroke survivor in setting goals was described by the teams as aligning with rehabilitation as usual, thus on the surface not requiring any change. This is supported by that fact that most stroke survivors responded that goals to a high degree (4–5/5) concerned important daily activities (28/30) and had been set together with the team (26/30), indicating that the component of person-centred goal setting was realised. Teams did however depict using the COPM differently to the way taught in the workshops. Examples of this was first setting goals according to their usual routine and only then proceeding to doing ratings using the scales of the COPM or doing the COPM for the purpose of the study only but having other goals on the side.

The intervention component of introducing the problem-solving strategy was mentioned directly by one team only. This team depicted striving to give stroke survivors ways of continuing rehabilitation on their own but felt that the focus on problem-solving strategies in the training workshops was insufficient. Data from the study specific survey revealed that a majority of stroke survivors (20/30) felt to a high degree (4–5/5) that they knew what to do on their own to recover from the stroke, but it is not clear whether the answers referred to strategies for performing daily activities or to physical exercise.

**Fig. 4:**
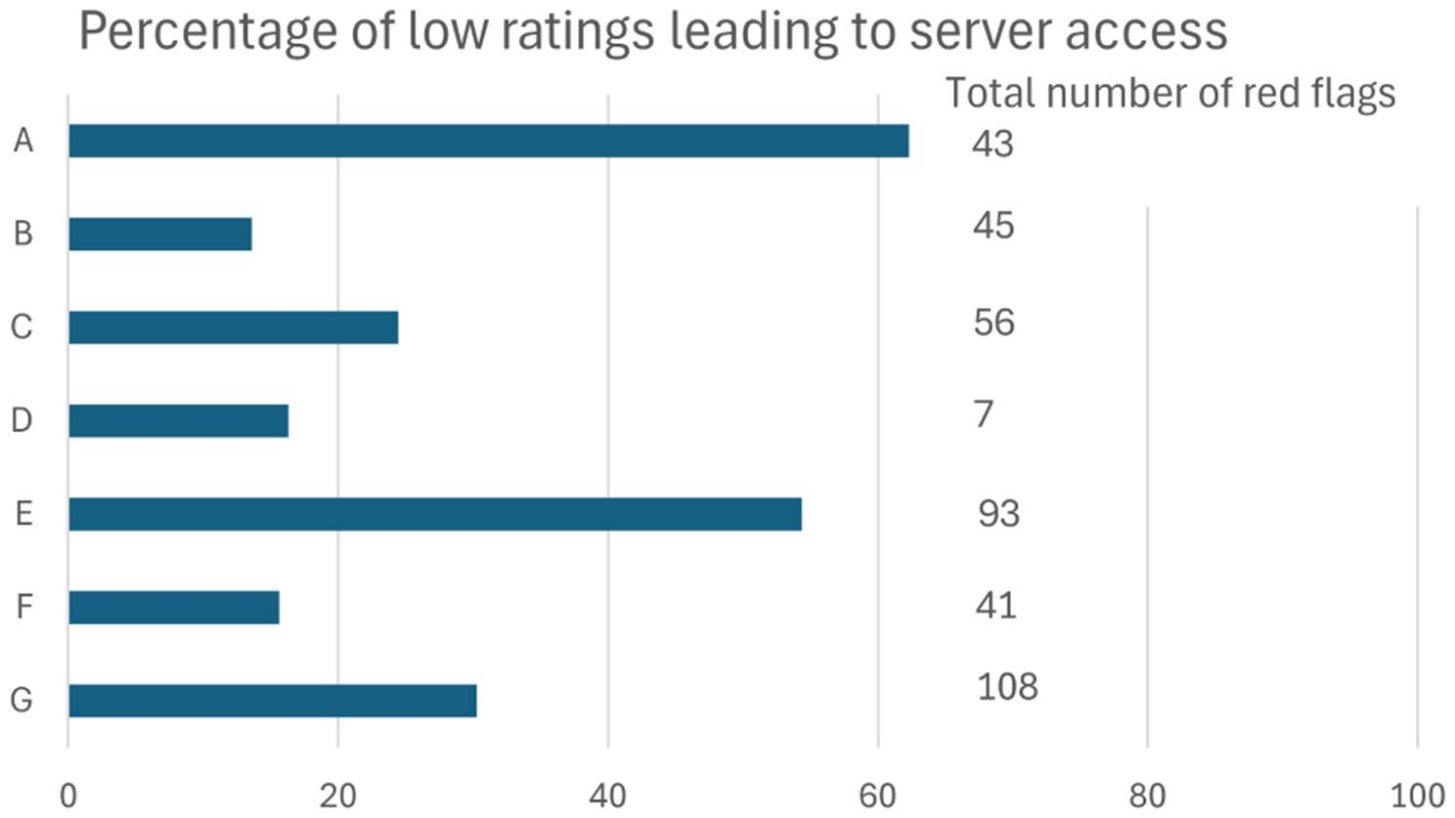
Percentage of low ratings leading to web server access per team

The teams were instructed to monitor goal achievement either through accessing the web server or through other contact with the stroke survivor, as low ratings could be interpreted as a need to renegotiate goals or strategies. In the process evaluation, this allowed for analysis of the frequency of server access as a response to low ratings. Response rate varied between teams with the least active team responding to 14% of low ratings by accessing the web server and the most active team responding to 62% of low ratings accessing the web server (see Fig. 4). Every low rating (1–2) or lack of reply resulted in a warning to the team via SMS, but no information is available on whether this led the team to take action.

Within F@ce 2.0, goals should be updated when needed (i.e. when stroke survivors score consistently high or low) and this occurred for five of the study participants. A few teams specifically mentioned not having updated the goals while others talked about accessing the web server being too time consuming or feeling that it was yet another task to fit into their busy schedule. Two teams did however mention having updated the goals with the stroke survivor but not changing them on the web server, meaning that the daily SMSs still contained the old goals. “*No*,* I don’t think I have [updated goals] but I can also say that I’ve had very few in the F@ce project….But the few that I have I haven’t updated anything.* “Team F.

In summary, possible fidelity issues were mainly related to the intervention components described as new to the teams, i.e. performing the COPM and acting on the daily ratings.

#### Adaptation

Two examples of adaptation were identified in the analysis. The first adaptation was carried out by one team only but relates to the recurring interview theme of finding it difficult to identify goals suited for daily reminders and evaluations. This team adapted the intervention by sending out the strategies and not the goals in the daily SMSs. The team felt that this adaptation strengthened the function of the daily ratings by making it clearer what to rate. The second adaptation was described by a team that typically referred stroke survivors to another unit before the end of the eight-week intervention. To be able to deliver F@ce 2.0 under these circumstances, the receiving team was provided with the necessary information to take over evaluation of the intervention, including further rehabilitation planning.

#### Reach

**Fig. 5:**
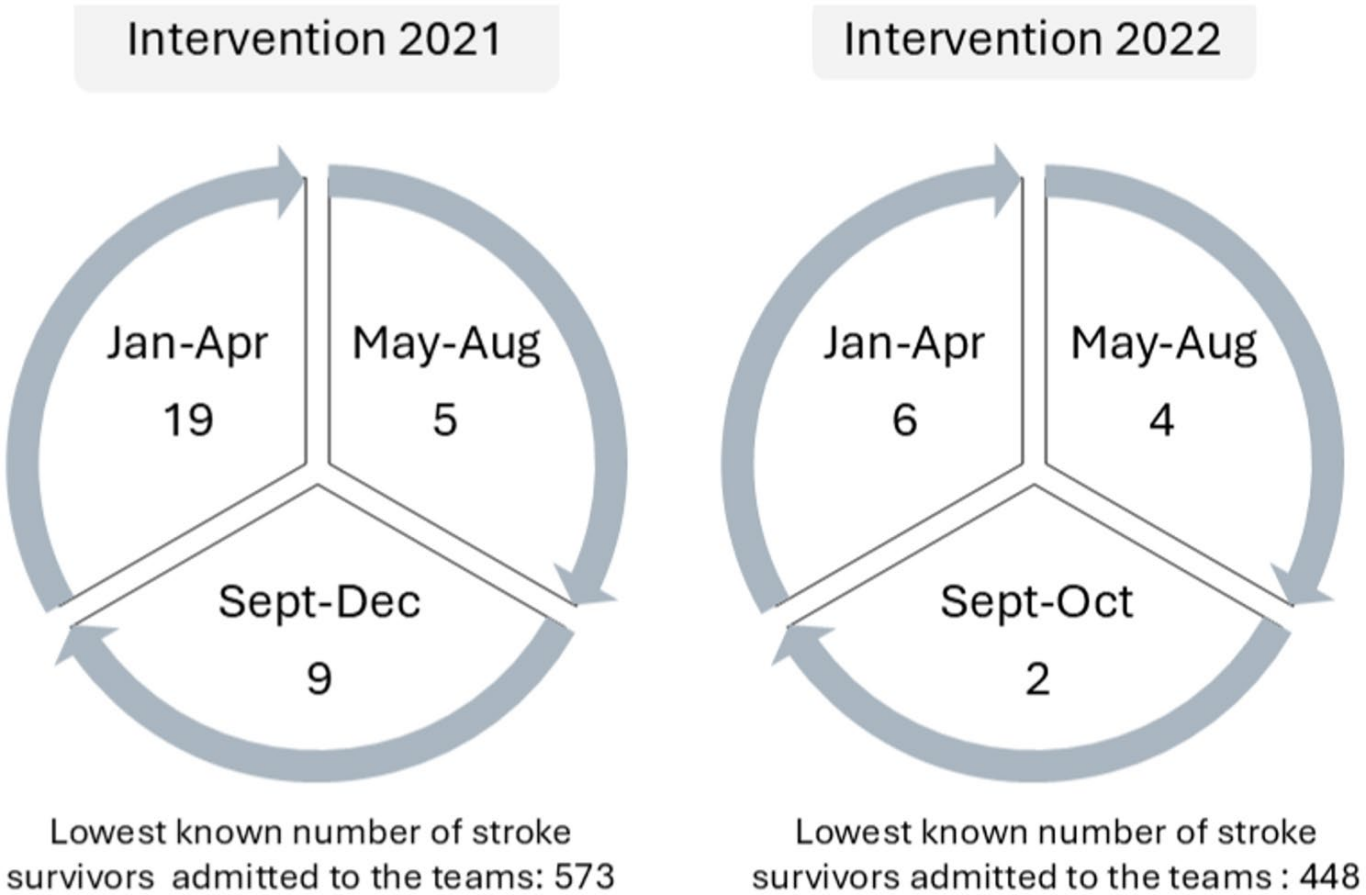
Recruitment flow. The lowest known number of stroke survivors admitted to the rehabilitation teams is based on data from teams’ statistic databases. Data were not available for all teams

Recruitment flow in F@ce 2.0 was slower than expected with most participants being included during the first tertile of 2021. Official records of the number of stroke survivors in contact with the team were not available for all units but a summation was conducted using data available. The recruitment flow is described in Fig. 5 below.

Analysis of the interviews and inclusion diaries revealed that teams often relied on additional considerations beyond the inclusion criteria when deciding whether to inform stroke survivors about F@ce 2.0. This was largely driven by a desire to avoid placing unnecessary burden on them. Hesitation to inform stroke survivors of the study was related to issues such as cognitive impairment, poor Swedish skills or the team thinking that SMSs would be too stressful for the patient. Some teams also discussed the timing being wrong with F@ce 2.0 coming in too early in the rehabilitation process or that the planned contact period would be too short to allow for evaluation. Further reasons were linked to the research study rather than the intervention per se. Teams reasoned that informing about F@ce 2.0 would be too much in relation to all other information given and that answering the questionnaires would be cumbersome for the stroke survivor.

*I had some who found it hard to go through all these papers that were sent. … This is like a big issue. How have you been thinking. There are like six-seven pages that a patient with a new stroke has to [fill out] and there can be a lot of cognitive impairment*. Team D

From the interviews, the teams’ view of the ideal candidate for F@ce 2.0 seemed to be someone who was relatively young or younger in the mind and who had a sufficient belief in their own ability to carry out training. Few stroke survivors seem to have fitted into that description, leading to a very limited number of potential participants being informed of the study.

#### Dose

Operationalising dose within the F@ce 2.0 intervention is complex due to the personalised character of the intervention. However, the stroke survivors’ daily ratings in response to the afternoon SMS was possible to measure and can be seen as an assessment of dose received. On average, the response rate per goal for all participants varied from 4.4 to 6.5 per team, indicating a high activity among most participants, although lower than the highest possible dose of 7 ratings per week.

## Rehabilitation teams’ experiences of delivering the intervention components

Research question 3, “What were rehabilitation teams’ experiences of implementing and delivering the intervention components” was included to inform refinement and future implementation of F@ce 2.0. To address this question, analysis explored rehabilitation teams’ experiences of delivering the respective components of the intervention.

### Person-centred goal setting focusing on daily activities

Teams described having had a focus on daily activities prior to implementation of F@ce 2.0, but some teams perceived that when using the COPM, goal setting became interdisciplinary, decreasing the risk of goals being set in professional silos. Teams also conveyed that goal setting based on the COPM lead to a qualitatively different result, capturing new aspects of daily life, particularly in terms of identifying activities that were personally relevant to the stroke survivor. Teams expressed that the stronger involvement of the stroke survivor in combination with an interdisciplinary perspective had led to the identification of unanticipated goals, such as wanting to spend more time with a spouse or wanting to be able to read to a grandchild:”*To me*,* using the COPM and rating was a bit like taking it to the next level. Because we started to try and capture what’s most important for the patient to work on. We can come up with a lot of suggestions of problems and goals but to sort of scout the terrain and see where the patient is*,* what’s important*.” Team G.

The teams’ experiences of the COPM were however not unequivocally positive. Using the COPM to rate performance and satisfaction was discussed as too complex for certain stroke survivors, especially when cognition was impaired. Teams further described that the COPM was perceived by some stroke survivors as too structured and thereby rigid. Only one of the teams reported continued use of the COPM after the F@ce 2.0. This team was the team with the highest reported degree of flexibility in terms of time.

### Negotiation of strategies to enable the chosen activities

Introducing Goal-Plan-Do-Check as a problem-solving strategy for stroke survivors was discussed in one interview only, revealing that the teams experienced inadequate preparation for delivering this intervention component.

*I don’t feel that we went through that in the workshop*,* this method of goal-plan-do-check. Maybe we thought that we were working like that already. It was nothing we went through specifically how to do anyway.* Team G

Another team mentioned having previously considered how to help stroke survivors in continuing rehabilitation on their own but did not feel that they had come any further in this.

### The ICT component of F@ce 2.0

#### Daily reminders to perform the chosen activities

A consequence of the daily reminders discussed by the team was that it forced them to be clearer in goal formulation so that reminders were comprehensible and goals were possible to rate. Guiding patients in this process was described as challenging, and there was an awareness about the balance between helping stroke survivors to break down a goal into smaller sub-goals and the risk of putting words in their mouths.

*It was a bit difficult with the goals. So that they would be*,* so they would receive text messages that felt good for the patient.* Team A

#### Daily self-evaluations

The feature of rating was highlighted in a multi-faceted way, both in relation to the COPM and to the daily evaluations. On the one hand, the rating was considered helpful since it gave the team a sense of both the importance of activities for stroke survivors and how they viewed their own performance. Rating was also perceived as a way for stroke survivors to follow their progress. On the other hand, the teams had observed that daily SMS ratings could be demotivating for stroke survivors with low awareness and low self-efficacy. The feasibility of using ratings in rehabilitation was questioned, particularly in the presence of cognitive impairment.

*So*,* we had a patient who rated the same for eight weeks on everything. But personally*,* he thought he had gotten better. Then you don’t know how he’s thinking when he’s rating*. Team E

Suggestions on how to resolve this included using the same scale for the daily ratings as in the COPM or to use a simpler scale, such as a traffic light or an indicator of having performed the activity at all, regardless of results. Even with these adjustments, a perceived barrier for collaborating through SMS communicated by the teams was that handling the technology could be too complicated for stroke survivors who sometimes needed help from spouses to handle the SMSs. Teams further expressed a view that not all rehabilitation goals were suited for daily ratings, for instance goals related to activities not typically performed every day or too overarching and long-term goals.

*That was something that we found a bit difficult in the beginning*,* that some goals are more long-term… not like something that can be evaluated every day* Team F.

The perception of the ICT component differed between teams with one team questioning its relevance due to a high intensity of personal contact with stroke survivors. Also, reviewing goals in person felt more natural compared to using the web server to check daily ratings. From the perspective of rehabilitation teams, getting notations about low ratings could be stressful, and one team even reported that the high frequency made warnings feel irrelevant.

## Discussion

This study contributed in-depth information about the process of implementing the F@ce 2.0 intervention within the clinical study setting. Analysis disclosed a high level of engagement among stroke survivors with the ICT component and revealed that rehabilitation teams perceived the utilisation of the COPM as leading to new types of rehabilitation goals. The analysis of implementation did, however, reveal low reach especially concerning more severe stroke, as well as fidelity issues related to inadequate implementation planning. Teams further reported feeling unprepared to deliver parts of the intervention. The aspects of context, implementation, and the proposed mechanisms of change related to the intervention components are strongly interconnected, and the following discussion will synthesise these findings.

The teams’ perception of inadequate preparation for implementing F@ce 2.0 may, in parts, be attributed to the limited contact between researchers and the team imposed by the COVID-19 outbreak. The importance of the training workshops, as well as accessible researcher-team contact, was highlighted in a previous feasibility study [22] but was not sufficiently accommodated during the forced transition to a remote format. Adapting training to an online format requires careful considerations with a strong focus on creating opportunities for interactivity [39]. Conducting the workshops online made it difficult to ensure that all team members participated actively. Since the F@ce workshops were delivered in an early phase of the pandemic, neither teams nor researchers were accustomed to the format, making it challenging for researchers to facilitate high levels of team engagement. To mitigate these barriers, assigning a local workshop facilitator could have been a strategy for enabling and encouraging discussions and ensuring the involvement of all participants [40, 41].

Although teams were given practical assignments between workshops to familiarise with the intervention components, they expressed a wish for more hands-on knowledge of the intervention. This aligns with the overall results from the feasibility study [23], although the finding that teams found it difficult to come up with rateable goals was new. Here, a suggested adaption of the intervention which warrants attention in future development was modifying it so that the daily ratings would pertain to the use of strategies to reach the goals rather than the level of goal fulfilment.

Although the COVID-19 pandemic affected the training workshops and the continuous contact between teams and researchers, analysis of team interviews indicate that some of the barriers to the implementation may have been present even with in-person workshops, especially the limited time to learn how to conduct the COPM and how to navigate the F@ce 2.0 web server. In relation to this, the teams proposed utilisation of case pedagogy, following a fictive stroke survivor throughout the whole intervention. The value of creating opportunities for interacting with an intervention and applying it to one’s context has been highlighted by previous research on education in healthcare [42]. Moreover, multimodal training can enhance knowledge and confidence in using new interventions [43].

It can be argued that implementing interventions within the context of an evaluation study entails inherent complexities. Firstly, both managers and teams were aware that the project was time-limited, potentially decreasing motivation to implement more thorough changes to clinical routines. Secondly, participation in the study meant undertaking the additional task of identifying potential participants. Concerning the first issue, a strength of F@ce 2.0 is that the intervention follows the typical process of rehabilitation, where assessment is followed by goal setting to formulate a plan [44]. On the surface, the intervention therefore seems well adapted to the delivery context. In practice, the intervention necessitated considerable changes to the clinical routine, in addition to the changes already made due to COVID-19. Reviews have identified lack of time as a systems-level barrier to the implementation of person-centred care [45] and to implementation within stroke rehabilitation [43]. Moreover, finding the right timing for introducing interventions has been emphasised [46]. New tools, such as the COPM for goal setting, need to be used consistently during a period before they become a part of routine practice and to be appropriately introduced to new staff [47].

The teams suggested appointing a team member with dedicated time to facilitate the implementation of F@ce 2.0. The role of an implementation facilitator can be understood as a bridge between the intended intervention and the local context of implementation and is a core concept of the Integrated Promoting Action on Research Implementation in Health Services (i-PARIHS) framework [48]. Facilitation is a complex task which requires dedicated time [49], and it has been suggested that more research is justified to identify how facilitators are best prepared [50]. Although a designated person from each team was appointed as a contact person, this was not equal to the role of an implementation facilitator, as they did not receive any extra time or training.

A further barrier for implementation was the low recruitment, limiting learning opportunities for the routinisation of the intervention. Previous research has highlighted that the constant time pressure of the clinical everyday life can led to the intervention being deprioritised [51, 52], a pattern that was described by the teams in the present study. Another underlying factor identified in previous reviews [51, 52] and mirrored by the present study was the strong ethics among rehabilitation teams related to protecting stroke survivors from unnecessary stress related both to study participation and to the intervention itself. Teams thus acted as gatekeepers, affecting both the recruitment rate and the reach of the intervention. Reach was influenced by teams’ perception of rating and mobile phone use as too complex for the intended population. The role of gatekeeping has been discussed in previous research and a suggested solution for preventing low recruitment despite the presence of eligible participants is to dedicate time to identify and discuss possible barriers before initiating recruitment [52]. In the F@ce 2.0 workshops, a specific focus on recruitment using facilitation techniques such as vignettes to spark discussion could have helped identify some of the issues expressed by healthcare professionals concerning both study participation and suitability of the intervention for stroke survivors [53]. A stronger stakeholder involvement in developing the intervention could also have prevented some of the issues raised by the team [19] and allowed for adaptation of the intervention prior to implementation. As an example, the idea of sending out strategies rather than goals could have been discussed. The low recruitment and reach also highlight the crucial role in implementation of actively involved leaders [54]. In the implementation of F@ce 2.0, managers received no specific instructions related to the implementation and some of them reflected on having been too detached from the intervention implementation.

 The hesitance among teams regarding the suitability of the intervention reflects research on the potential digital divide affecting stroke survivors, where impaired fine motor skills and dysarthria [55], aphasia [56] and cognitive impairment [57] have been identified as barriers to technology use. Digital health technology may also be perceived as less relevant compared to other uses [58], with a preference for personal contact [59]. Previous exploration of stroke survivors’ experience of participating in the F@ce 2.0 intervention revealed that the ICT component had the potential to increase motivation, but that even this relatively simple technology could be challenging to use [26]. The degree of completed daily ratings in the present, indicates that most stroke survivors were able to use the technology, although, a majority of participants had a mild stroke. SMS technology offers the advantage of relying on an established reminder function within the Swedish healthcare system, which may reduce barriers to broader implementation.

### Methodological considerations

One aim of this process evaluation was to assess fidelity and dose of F@ce 2.0. Measuring fidelity in complex interventions requires ample planning and researcher time, and it is recommended that some fidelity measures be built into the intervention [60]. A strength of F@ce 2.0 is therefore that the web server could be used to gather fidelity information. A limitation of this study is, however, that no pre-determined criteria were established for fidelity in terms of the number of goals updated or for dose (number of daily ratings) [61]. Furthermore, no observational data was obtained to ensure that the intervention was delivered as intended.

Using focus group interviews [62] with rehabilitation teams to explore their perception of delivering the intervention and of how intervention components were being realised provided a valuable inside perspective. Although several critical reflections were expressed in the interviews, a limitation of the study is that team members may have been reluctant to share their shortcomings in delivering the intervention in a group setting [63]. Furthermore, teams could only dedicate a limited time to interviews. During the analysis of the interview material, several follow-up questions arose, indicating that additional interview material or observations would have been beneficial. Conducting more than one interview with the teams would have allowed for a deeper exploration, but it is unlikely to have been feasible without some financial reimbursement.

## Conclusions

This process evaluation revealed that although rehabilitation teams acknowledged the potential of F@ce 2.0, implementation was limited, and the reach within the intended patient population was low. A key area for improvement was the limited contact with the rehabilitation teams, both in terms of support to teams and in terms of monitoring implementation outcomes. The implementation would likely have gained from involvement of the rehabilitation teams at an earlier stage to better identify both educational needs and organisational prerequisites.

## Electronic Supplementary Material

Below is the link to the electronic supplementary material.

Supplementary Material 1: Supplement 1 – Interview guides

Supplementary Material 2: Supplement 2 – Survey to stroke survivors

Supplementary Material 3: Supplement 3 - Analysis frame according to the MRC guidance for process evaluation

Supplementary Material 4: Supplement 4 – Mapping of results to the CFIR

Supplementary Material 5: Supplement 5 – Table of implementation outcomes

## Data Availability

The raw data for this study includes transcripts of interviews which could potentially lead to identification of individuals. Since the interviews include sensitive information, we are unable to share these data publicly due to ethical concerns. Relevant, de-identified excerpts of the transcripts are included in the paper. Quantitative data is available upon reasonable request.
