# Supplementary material for: Process evaluation of F@ce 2.0, a team-based, person-centred intervention for rehabilitation after stroke supported by ICT
Source: BMC Health Serv Res. 2026 May 7;26:663. doi: 10.1186/s12913-026-14628-6 (PMC13154473; doi:10.1186/s12913-026-14628-6)
Supplement: Supplementary file 1 — Supplementary Material 1: Supplement 1 – Interview guides [file 12913_2026_14628_MOESM1_ESM.pdf]

Interview 1 with teams (during the first 4 months)

Purpose:

To examine the implementation process within the teams, the effectiveness of the workshops in supporting staff in using F@CE, and the impact of factors connected to the team on this process.

1. If you were to tell someone what F@ce is, what would you describe that F@CE means and what you do? *(means for you as staff, for the patient, relatives)*

2. Using F@CE, is it different from your usual way of working – and if so, how?

3. You attended workshops that were meant to prepare you to use F@ce in your work – now that you have used F@ce – tell me how you think the workshops worked to prepare you for using F@ce?

   (Content: what were the essential parts of WS? What was less rewarding? What - if anything - was missing?)

   (Form: How did it work that it was digital? Meeting another team during workshops? To be able to reflect/discuss together? Could it have been done better in another way – how?)

4. If we were to do this again (workshop), what do you think we would need to think about then? Is there something that we should do more or less of? What would a dream scenario look like to make you prepared. What would be ideal for you?

5. Can you tell us how starting to use F@ce has worked in your daily work? Have there been any challenges/strengths – and if so, which ones?

   (enough knowledge/training, equipment that works, support from managers and colleagues, time, motivation to initiate new ways of working, to make it fit with other routines/ways of working, prevailing values and priorities, technology experience, to make it fit with the patients, How do you feel about the support from the research group now that you have started to use F@ce?...)

6. Do you use any of the components/ways of thinking in F@ce for other patients? If so, what? What *is the best thing about using F@ce?)*

7. Do you get questions from your colleagues and what do you tell them about F@ce?

Interview 2 with teams (after inclusion of the last stroke survivors)

1. **Tell me about the stroke rehabilitation here** – (which patients come to you, how they come to you, how long you see them, if they go on to another unit later and so on, how do you plan for each patient, how do you set goals)

2. **Tell me how you have worked with F@CE – start with the first meeting with the patient and tell me what you have done and how you have done it**

    - *Let the participants tell their stories freely and use the points below as support if necessary to request more details*

    - *Follow up if necessary with questions about "who" did (has the whole team been involved or just AT/FT) and in-depth "how" questions*
    - *If the participants say that something has been done "differently" or parts have been skipped,*
      -Follow up and ask if this has been done with all patients or if it has been adapted to only one patient

      -Why the adaptation was made, and how the reasoning went

    - At the first meeting
    - To carry out an activity together with the patient/ film
    - To go through and assess the activity together with the patient, possibly relatives
    - To formulate 3 goals based on COPM.
    - Working from GOALS – PLAN – TRAIN – RECONCILE
    - Registering in the server and entering the server
    - To inform everyone in the team / relatives/home care
    - To carry out training with strategies from study 1, 2 and 3, GOAL-PLAN-TRAIN-CHECK and theoretical frames of reference such as client-centeredness.
    - To evaluate together with the patient and any relatives and possibly reformulate goals and set new goals

3. **Tell me how F@CE worked as a person-centred (client-centred) way of working** (specific components you see as person-centred/not person-centred, how, why.....),

4. **Tell me about your experiences of F@CE as a tool in the work with patients** (about using COPM to set goals, about how it has been received and used by patients, difference over time in patient engagement, if and how you have taken part in and used the daily ratings/goals, updated goals, looked at the server sometimes/regularly, if it has been supportive/motivating/positive – how/why, difficult/hindering/not made any difference – how, why).

5. **Tell me about your experiences of F@CE and relatives** (about what the role of relatives has been, how F@CE been received by relatives, what relatives' participation/support for their loved one with stroke looked like, how (if) it has been supportive/motivating/positive – how/why, difficult/hindering/not made any difference - how, why)

6. **Tell me about your reasoning when you asked patients to try F@CE and participate in the study** (about which patients you asked, which ones you did not ask, about how relatives influenced, could you have asked more patients, which patients are F@CE suitable for... how, why). *In the first interview not wanting to burden relatives with having to help with F@ce was mentioned. Follow-up on this.*

7. **Tell me how F@CE worked for you as a staff** (in the team's work as a team, how the team worked during this time, how F@CE worked in the teamwork, any impact, practically/technically, difficulties, things that worked well, if any other preparation had been needed, why/how) *Some difficulties were described in the first interviews – remember to capture what it was like at the end – has it become easier over time?*

8. **You told me before about how stroke rehabilitation works – tell us what it has looked like when you used F@CE – has anything changed (**fewer visits...?)

9. **Tell me if and how the use of F@CE has affected how you work with other patients** (teamwork, how goals are set, ...)

10. **Tell me how you have done when new staff have been hired (**have they been introduced to F@CE and the study – how?)

11. **If you were to continue to use F@CE, how would you use it? What would you do differently?**

12. **Something that I haven't asked about that you want to tell me?**

Managers

1. **Tell me about your role and your mission (**how long you have been a manager here, your assignment).

2. **Tell me about stroke rehabilitation here** – (about your team's role in the stroke care chain – the team's mission, which patients come to you, what determines which interventions patients receive, which professions are included in the home rehab team – as a "basis" and as consultants)
    a. How many patients with stroke per year?
    b. How many visits by the home rehab team on average?

3. **Tell me your perception of how F@CE is supposed to work**

4. **Tell me about the contact you have had with F@CE and how you have been involved** (involved in the decision to participate in the study, supported the staff in the use of F@CE, supported the staff in participating in the study...)

5. **Tell me how you perceive that F@CE has been integrated into the work of your staff** (how it has fit into the stroke care chain, if you have changed anything in your way of working, how it has worked with the patients you have, any obstacles you have encountered, how it has worked in the teamwork, change over time, learning culture and receptivity to new ideas and ways of working, other changes that have had an impact, if it has demanded more or less time from the staff).

6. **Tell me how you perceive that the workshops and support/collaboration with the researchers have worked** (if you participated in the workshop, if you have received any feedback from the staff)

7. **Tell me about the procedure when new staff have been hired (**have they been introduced to F@CE and the study – how?)

8. **Tell me if you have received for any feedback on how F@CE worked for the staff and for patients** (something difficult, complicated – how/why?, something that has been perceived as supportive/positive – how/why?)

9. **If you were to continue to use F@CE, how would you use it?** (what would you do differently – why, other patient groups, suggestions for further development or adaptation, what would it be like if F@CE were on the existing IT platform)

10. **If you are thinking about a future use of F@CE, tell me how it would work in practice and what possible changes are needed linked to reimbursement systems and IT systems etc** (what does the reimbursement look like today, reimbursement for visits/telephone, reimbursement for extra-long initial visits to do COPM, cost of SMS reminders (overhead or individual clinics), extra visits to formulate new goals if necessary, technical resources, stand-alone or integrated into 1177)

11. **Tell me what participation in the F@CE study has meant for your unit going forward** (some experiences/lessons you will take with you)

12. **During the study period, from January 2021 until now – has anything happened in yur unit that affected the work - how**?(organizational changes, downsizing...)

13. **What has the staff situation been like during the period in question?** (how staffing may have affected the use of F@CE and participation in the study)
