# Supplementary material for: Process evaluation of F@ce 2.0, a team-based, person-centred intervention for rehabilitation after stroke supported by ICT
Source: BMC Health Serv Res. 2026 May 7;26:663. doi: 10.1186/s12913-026-14628-6 (PMC13154473; doi:10.1186/s12913-026-14628-6)
Supplement: Supplementary file 2 — Supplementary Material 2: Supplement 2 – Survey to stroke survivors [file 12913_2026_14628_MOESM2_ESM.pdf]

Questions About Training/Rehabilitation Related to Your Stroke

1. To what extent have you and the rehabilitation team set goals for your rehabilitation together?

- ☐ 5 – To a very great extent
- ☐ 4
- ☐ 3
- ☐ 2
- ☐ 1 – Not at all
- ☐ Don't know
- ☐ Not applicable, I have no goals for the training/rehabilitation

2. To what extent are your rehabilitation goals related to activities that are important in your everyday life?

- ☐ 5 – To a very great extent
- ☐ 4
- ☐ 3
- ☐ 2
- ☐ 1 – Not at all
- ☐ Don't know
- ☐ Not applicable, I have no goals for the training/rehabilitation

3. Can you describe/mention some of the goals for your training/rehabilitation?

Goal 1:

____

____

____

____

Goal 2:

____

____

____

____

Goal 3:

____

____

____

____

4. To what extent do you know how to train on your own to recover after your stroke?

- ☐ 5 – To a very great extent
- ☐ 4
- ☐ 3
- ☐ 2
- ☐ 1 – Not at all
- ☐ Don’t know
- ☐ Not applicable to train on my own

5. To what extent do you receive support from your relatives in your training/rehabilitation?

- ☐ 5 – To a very great extent
- ☐ 4
- ☐ 3
- ☐ 2
- ☐ 1 – Not at all
- ☐ Don’t know
- ☐ Not applicable to receive support from relatives

6. During a typical week, how many days do you carry out training/rehabilitation on your own?

- ☐ Every day
- ☐ Most days
- ☐ A few days
- ☐ Never

7. If you train on your own, approximately how much time do you spend on training/rehabilitation on a typical day?

- ☐ 0–30 minutes
- ☐ 30–60 minutes
- ☐ 1–2 hours
- ☐ More than 2 hours
