# Supplementary material for: Process evaluation of F@ce 2.0, a team-based, person-centred intervention for rehabilitation after stroke supported by ICT
Source: BMC Health Serv Res. 2026 May 7;26:663. doi: 10.1186/s12913-026-14628-6 (PMC13154473; doi:10.1186/s12913-026-14628-6)
Supplement: Supplementary file 5 — Supplementary Material 5: Supplement 5 – Table of implementation outcomes [file 12913_2026_14628_MOESM5_ESM.pdf]

Supplement 5

Overview of Implementation Outcomes

**Fidelity**		
**Operationalisation**	**Assessment**	**Result**
Stroke survivors' perception that goals concerned important daily activities.	Rated on a scale from 1 (not at all) to 5 (to a very high degree.	28/30 stroke survivors reported that goals to a high degree (rating of 4 or 5) concerned important daily activities.
Stroke survivors' perception of goal setting as collaborative.	Rated on a scale from 1 (not at all) to 5 (to a very high degree.	26/30 stroke survivors reported that goals to a high degree (rating of 4 or 5) had been set together with the team.
Use of COPM.	Qualitative interviews	COPM not conducted according to the manual
Introduction of general problem-solving strategy.	Qualitative interviews	Lack of data. One team reported that this themes was not covered in the workshops.
Stroke survivors' perception of knowing what to do on their own to reach the goals.	Rated on a scale from 1 (not at all) to 5 (to a very high degree.	28/30 stroke survivors reported knowing to a high degree (rating of 4 or 5) what to do on their own to reach the goals.
Serve access in response to stroke survivors rating low goal fulfilment.	Data from the F@ce 2.0 web server on percentage of low ratings leading to teams accessing the server.	Variations between teams.<br>Lowest level: 14%<br>Highest level: 62%
Goals updated when needed.	Number of participants where new goals were registered on the F@ce 2.0 web server during the intervention.	5
Goals updated when needed.	Qualitative interviews	Report of goals being updated in dialogue with the patient but not changed on the server.
**Adaptation**		
**Operationalisation**	**Assessment**	**Result**
Examples of adaptations reported by the teams.	Qualitative interviews	Sending out the strategies and not the goals in the daily SMSs to stroke survivors.

		Evaluation conducted by the team to which the patient is referred.
**Reach**		
**Operationalisation**	**Assessment**	**Result**
Percentage of stroke survivors seen by teams and reach by the intervention.	Process data from teams.	Inconclusive due to lack of process data from teams. Indication of very low reach.<br>**2021: 6%**<br>Included: 33<br>Lowest known number of stroke survivors seen by the teams: 573<br>**2022: 3%**<br>Included: 12<br>Lowest known number of stroke survivors seen by the teams: 448
Factors outside exclusion criteria mentioned by teams as reason for not informing patients about F@ce 2.0.	Qualitative interviews	Fatigue<br>Cognitive impairment<br>Poor Swedish skills<br>Old age<br>Risk for stroke survivors being stressed by participating in a research study
**Dose (e.g. dose received)**		
**Operationalisation**	**Assessment**	**Result**
Stroke survivors' response rate to daily ratings.	Data from the F@ce 2.0 web server on percentage of ratings performed. Analysed as average per team	4.4-6.5/7 of ratings performed.<br>(Maximum of 1 rating/goal/day = 7 ratings per week)
